# Robotic single‐site versus multiport radical hysterectomy in early stage cervical cancer: An analysis of 62 cases from a single institution

**DOI:** 10.1002/rcs.2255

**Published:** 2021-05-07

**Authors:** Tae‐Kyu Jang, Hyewon Chung, Sang‐Hoon Kwon, So‐Jin Shin, Chi‐Heum Cho

**Affiliations:** ^1^ Department of Obstetrics and Gynecology Keimyung University School of Medicine Daegu South Korea

**Keywords:** early stage cervical cancer, robotic multiport radical hysterectomy, robotic single‐site radical hysterectomy

## Abstract

**Background:**

This study aimed to compare the surgical outcomes and cost of robotic single‐site radical hysterectomy (RSSRH) versus robotic multiport radical hysterectomy (RMPRH) with pelvic lymph node dissection in early stage cervical cancer.

**Methods:**

Sixty‐two patients with early stage cervical cancer were recruited between November 2011 and July 2017 and underwent RSSRH (20 patients) and RMPRH (42 patients) for early stage cervical cancer using the da Vinci Si Surgical System (Intuitive Surgical).

**Results:**

There were no significant difference between the two groups in most of parameters. However, postoperative hospital discharge and total hospital costs for RSSRH were significantly shorter than RMPRH (both *p* < 0.001). However, lymph node retrieval of RMPRH was significantly higher than RSSRH in (18.0 vs. 9.5, respectively; *p* < 0.001).

**Conclusions:**

RSSRH has comparable surgical outcomes to the RMPRH method. RSSRH could be considered a surgical option in a well‐selected patient group.

## 1 INTRODUCTION

Cervical cancer is a preventable disease primarily due to its long pre‐invasive state, availability of cervical cytology screening programs and effectiveness of the treatment of pre‐invasive lesions. The morbidity and mortality of cervical cancer has significantly decreased in developed countries due to the availability of efficient and accessible screening programs as well as diagnostic and treatment facilities.^1^ However, cervical cancer is still the second most common cancer in women according to the World Health Organization, with an estimated 530 000 new cases and 310 000 deaths annually.^2^

The standard surgical treatment for early stage cervical cancer with no intent to preserve fertility is radical hysterectomy with pelvic lymph node dissection (PLND).^3^, ^4^ The methods used for radical hysterectomy are laparotomy, and minimally invasive surgery (MIS), including conventional laparoscopy or robot‐assisted laparoscopy. MIS has several advantages over laparotomy, including reduced pain, improved aesthetics, shorter length of hospital discharge and faster return to normal activities.^5^, ^6^ Laparoendoscopic single‐site surgery (LESS) is one of the newest innovations in MIS and has several potential applications in gynaecologic oncology. Despite the more advanced surgical methods, LESS presents various surgical challenges, including a limited range of motion due to the parallel angle of surgical instruments, difficulty in manipulating a flexible camera and positioning of surgical instruments in a limited space through a small skin incision.^7^, ^8^

To overcome these surgical challenges, robotic single‐site surgery (RSSS) platforms have been developed recently. RSSS, which is one of the best advanced forms and an FDA‐approved alternative to LESS, emerged in 2013 as an attempt to (1) combine easier manipulation; (2) improve a magnified three‐dimensional view; and (3) provide wider range of motion with wristed instruments acquired with robotic technology using a single skin incision.^9^, ^10^ However, there is still a lack of studies addressing the surgical outcomes of robot‐assisted operations, since robot‐assisted radical hysterectomy is a relatively novel surgical technique and its surgical outcomes have not been investigated in randomised controlled trials.^11^ The aim of the study was to compare the surgical outcomes and total costs of robotic single‐site versus multiport radical hysterectomy with PLND in early stage cervical cancer.

## 2 MATERIALS AND METHODS

### 2.1 Patients and basic characteristics

A total of 62 patients who underwent robot‐assisted radical hysterectomy between November 2011 and July 2017 in the Department of Obstetrics and Gynecology at the Keimyung University Dongsan Medical Center (Daegu, South Korea) were included in this study. This retrospective cohort study was approved by the Institutional Review Board of Dongsan Medical Center (IRB 2017‐11‐026). Prior to their operations, all patients were informed about robot‐assisted radical hysterectomy techniques, benefits and risks of laparoscopic or laparotomic conversions and provided signed consent forms.

The stages of cervical cancer were classified according to the International Federation of Gynecology and Obstetrics (FIGO) classification revised in 2018.^12^ Patients with a preoperative diagnosis of early cervical cancer (FIGO stage IA1 with lymphovascular space invasion, IA2 and IB1) were selected. There was no specific contraindication to robot‐assisted radical hysterectomy operation, except for the following conditions: (1) evidence of metastasis to other organs in preoperative imaging and (2) high‐risk pathology (ex. neuroendocrine tumour) on preoperative cervical biopsy. Although there were no restrictions for patients related to body mass index (BMI) or previous abdominal surgeries, robotic multiport radical hysterectomy (RMPRH) was performed in patients with a BMI of 30 or higher or a history of previous surgery with high probability of intrapelvic adhesion.

The total operative time was subdivided as follows: (1) preparation time (the time from the first incision to the end of port replacement); (2) docking time (the time from insertion of the robotic arms through trocars to introduction of the robotic instruments); (3) console time (the real surgical time, measured from the first manipulation by the surgeon to the last manipulation to repair the vaginal cuff); and (4) closure time (the time from release of docking to finishing the skin suture). The total operation time was calculated from the preparation time to closure time. Intraoperative parameters included estimated blood loss, requirement for blood transfusion, conversion to multiport laparoscopy or laparotomy and intraoperative complications. Postoperative parameters included length of hospital stay, total hospital charge, haemoglobin change, lymph node retrieval and postoperative complications.

### 2.2 Surgical technique

Each patient was placed in a typical low lithotomy position after induction of general anaesthesia. The body of the patient was then positioned in the Trendelenburg position (at a 30° angle). To prevent venous thrombosis after robotic radical hysterectomy, intraoperative automatic compression devices were installed on both lower extremities of the patient. After general anaesthesia, a Foley catheter was inserted into the bladder, and the vaginal cavity was sterilised with povidone‐iodine solution. A Rumi® uterine manipulation device (Cooper Surgical) was inserted to hold the cervix tight and enable efficient movement during the operation. All cases were performed using a da Vinci Si® Surgical System (Intuitive Surgical). The surgical team consisted of the primary surgeon, bedside assistant and robot system dedicated scrub technician and circulating nurse. Sixty‐two cases of robotic radical hysterectomy were performed by two gynaecologic surgeons experienced in robotic surgery. After completion of the RSSRH and RMPRH robotic settings, all robotic radical hysterectomy operations were performed sequentially from bilateral pelvic node dissection to Type II or III hysterectomy (classification of radical hysterectomy by the Surgeons Committee of the Gynecologic Cancer Group, which was part of the European Organization of Research and Treatment of Cancer in 2007) according to the patient's age, preoperative pelvic examination and imaging findings.^13^ The resected uterus and adnexa were removed through the vagina, and the vagina cuff was repaired with a continuous suture by V‐LocTM (Covidien), which is a unidirectional barbed suture with a curved needle in all cases.

### 2.3 Robotic single‐site radical hysterectomy

This single‐site instrument is a multiple‐channel single port composed of a robotic, 8.5‐mm, high definition with a three‐dimensional endoscope, two types of curved robotic cannulas and one 5‐mm accessory cannula. A single 2.5‐cm vertical periumbilical incision was usually made to the left of the umbilicus, performed using an open Hasson approach. The lubricated single‐site port was then inserted into the abdominal cavity, and the lower rim of the single‐site port was clamped using atraumatic Kelly forceps. After checking the other organs, pneumoperitoneum was made at a pressure of 12 mmHg with carbon dioxide. A trocar for the camera and a three‐dimensional, 8.5‐mm endoscope (30°) were inserted carefully along the endoscopic cannula and the abdominal cavity was inspected to confirm the feasibility of the RSSRH operation. The position of the da Vinci robotic body was situated between the widened patient's feet. One 5 × 250‐mm curved cannula (Arm 2) was inserted through the designated lumen until the end line of the cannula was visible in the field of endoscope vision. While the other cannula (Arm 1) was inserted using the same method, the already inserted cannula was held by the assistant to prevent displacement. Finally, two curved cannulas were positioned in cross position to avoid collision, and then a monopolar hook (Arm 2) and bipolar fenestrated bipolar grasper (Arm 1) were placed in each arm of the cannulas for the right‐handed surgeon.

The assistant's 5‐mm accessory cannula was inserted to perform several functions in the procedure^1^: suction and irrigation^2^; coagulation and cutting simultaneously by the LigaSure 5‐mm blunt tip (Covidien)^3^; and insertion of V‐LocTM 2‐0 sutures (Covidien), which is a unidirectional barbed suture used exclusively with a straightened needle.

### 2.4 Robotic multiport radical hysterectomy

In the RMPRH, a 12‐mm trocar was placed at 5 cm cranial to the umbilical level after the creation of a pneumoperitoneum to 12 mmHg with a transumbilical veress needle. Three 8‐mm trocars, specific for the da Vinci robotic systems (Intuitive Surgical) were placed: one (Arm 1) on the left side of the abdominal wall, medial and cranial to the left anterior upper iliac spine and two on the right rim of the abdominal wall. The first (Arm 3) on the right lowest side and the second (Arm 2) medial and cranial to the right anterior upper iliac spine on the equal line of the left trocar (Arm 1) were placed. An assistant 10‐mm trocar was located on the left lowest side of the pelvic wall, 7–10 cm laterally, from Arm 1. After we positioned the Trendelenburg place (30°), the da Vinci robotic column was placed near the operating table between the patient's feet and docked. The following instruments were introduced: a bipolar grasper and a PK grasper on the right robotic trocars (Arms 2 and 3, respectively), and a monopolar scissor on the left robotic trocar (Arm 1). A 30° Surgical Intuitive endoscope was used during all operations.

### 2.5 Statistics

The data collected from hospital medical records were reported as median or percentages for continuous and categorical variables, respectively. Differences between the RSSRH and RMPRH groups were tested using the *χ* ^2^ test, *t* test and analysis of covariance (ANCOVA) tests for categorical and continuous variables, respectively. *p*‐values of less than 0.05 were considered statistically significant. Data were analysed using SPSS version 19.0 (SPSS).

## 3 RESULTS

The basic characteristics of the patients are shown in Table 1. Overall, the descriptive characteristics of the two groups were similar. The median age of patients in the RSSRH group was higher than RMPRH, but the difference was not statistically significant (50.5 vs. 46.0 years, respectively; *p* = 0.13). In both groups, a history of caesarean section was reported by approximately 30% of patients (30.0% in RSSRH; 35.7% in RMPRH), but there were no cases of conversion to either laparoscopy or laparotomy in both groups. The most common histologic type was squamous cell carcinoma (65% in RSSRH; 66.7% in RMPRH) and the most common FIGO staging was IB1 (80% in RSSRH; 76.2% in RMPRH).

**TABLE 1:** Basic characteristic of patients

Parameter	RSSRH (*N* = 20)	RMPRH (*N* = 42)	*p*
Age (years), range	50.5 (32–65)	46.0 (32–67)	0.133
Body mass index (kg/m^2^), range	22.9 (19.4–32.0)	23.2 (16.2–33.3)	0.061
Previous abdominal surgery			0.741
No	14 (70.0)	27 (64.3)	
Yes	6 (30.0)	15 (35.7)	
Parity			0.623
0	2 (10.0)	4 (9.5)	
1	1 (5.0)	9 (21.4)	
>2	17 (85.0)	29 (69.0)	
Histology			0.892
Squamous cell carcinoma	13 (65.0)	28 (66.7)	
Adenocarcinoma	6 (30.0)	11 (26.2)	
Adenosquamous cell carcinoma	1 (5.0)	3 (7.1)	
FIGO staging			0.216
IA1	1 (5.0)	1 (2.4)	
IA2	3 (15.0)	9 (21.4)	
IB1	16 (80.0)	32 (76.2)	

*Note:* Data are presented as number (%) or median.

Abbreviations: FIGO: International Federation of Gynecology and Obstetrics in 2018; RMPRH: robotic multiport radical hysterectomy; RSSRH: robotic single‐site radical hysterectomy.

The operative outcomes of the patients are shown in Table 2. Overall, there was no statistical difference between parameters associated with intraoperative outcomes and there were no major intraoperative complications in either groups. The median total operation time was 186.0 min (range, 128–259 min) and 194.0 min (range, 138–259 min) in the RSSRH and RMPRH groups, respectively (*p* = 0.1).

**TABLE 2:** Operative outcomes of patients

Parameter	RSSRH (*N* = 20)	RMPRH (*N* = 42)	*p*
<Intraoperative>			
Estimated blood loss (ml)	215.0 (50–500)	221.4 (100–500)	0.911
Blood transfusion	1 (4.2)	3 (7.1)	0.759
Conversion to laparoscopy or laparotomy	0	0	–
Major intraoperative complication^a^	0	0	–
Operation time (min), median			
Docking time	6.5 (4–14)	10.0 (3–20)	0.107
Console time	102.5 (51–158)	117.5 (63–255)	0.112
Closure time	25.0 (13–45)	25.0 (10–50)	0.471
Total	186.0 (128–259)	194.0 (138–329)	0.1
<Postoperative>			
Major postoperative complications^b^	1 (5.0)	2 (4.8)	0.967
Haemoglobin drop (g/dl)	1.3 (0.3–2.5)	1.5 (0.2–4.0)	0.224
Lymph node retrieval	9.5 (4–17)	18.0 (3–36)	<0.001
Postoperative hospital discharge (days)	6.0 (4–14)	11.0 (4–27)	<0.001
Total hospital charge (won)	6323 422	9158 426	<0.001

*Note:* Data are presented as number (%) or median (range).

Abbreviations: RMPRH: robotic multiport radical hysterectomy; RSSRH: robotic single‐site radical hysterectomy.

^a^ Major intraoperative complication includes hemodynamically unstable vital signs including massive bleeding, and other organ injury which require cooperation surgery.

^b^ Major postoperative complication includes hernia, bowel injury or ileus, vaginal cuff dehiscence and infection and vaginal bleeding which requires surgical intervention or hospital re‐admission.

There were no statistical differences in major postoperative complications and haemoglobin change between the two groups. Postoperative complications included one case of re‐admission due to vaginal cuff infection in the RSSRH group which occurred 4 weeks after surgery. There were two complications in the RMPRH group: one patient was treated conservatively with small bowel ileus and one patient with vaginal dehiscence. However, there was a significant difference in several parameters. The median retrieval of pelvic lymph nodes was 9.5 nodes (range, 4–17 nodes) in the RSSRH and 18.0 nodes (range, 3–36 nodes) in the RMPRH group (*p* < 0.001). The median postoperative hospital discharge was also different between the two groups (*p* < 0.001) as it was 6 days (range, 4–14 days) and 11 days in (range, 4–27) the RSSRH and RMPRH groups, respectively. The median total hospital charge was 632.3 (10 000 won) (range, 439–776) in the RSSRH group, and 915.8 (10 000 won) (range, 547–1359) in the RMPRH group (*p* < 0.001).

Oncological outcomes between the two surgical methods were also explored and these were not statistically different (Table 3). The median follow‐up time was 38 months. There was no significant difference between the two groups in the risk factor related to recurrence in postoperative biopsy. Adjuvant therapy was confirmed in approximately 30% in both groups, and recurrence was confirmed in one patient in the RSSRH group and in two patients in the RMPRH group. All three patients with recurrence died, and one of them who underwent RMPRH died due to small bowel perforation during chemoradiation.

**TABLE 3:** Oncologic outcomes of patients

Parameter	RSSRH (*N* = 20)	RMPRH (*N* = 42)	*p*
Postoperative biopsy			
Tumour size (mm), range	16.2 (8–25)	18.5 (6–27)	0.263
Lymphovascular space invasion	7 (35.0)	10 (23.8)	0.377
Lymph node metastasis	0 (0.0)	2 (4.8)	0.556
Parametrium metastasis	1 (5.0)	4 (9.5)	0.663
Adjuvant therapy	7 (35.0)	13 (31.0)	0.75
Radiation	1 (5.0)	1 (2.4)	
Chemoradiation	6 (30.0)	12 (28.6)	
Recurrence	1 (5.0)	2 (4.8)	0.967
Alive	19 (95.0)	40 (95.2)	0.967
Death	1 (5.0)	2 (4.8)	
Due to disease	1 (5.0)	1 (2.4)	
Related to disease	0 (0.0)	1 (2.4)	

*Note:* Data are presented as number (%) or median (range).

Abbreviations: RMPRH: robotic multiport radical hysterectomy; RSSRH: robotic single‐site radical hysterectomy.

Tables 4, 5, 6 are re‐verified by using the ANCOVA test for statistical differences in Table 2 such as postoperative hospital discharge, total hospital charge and lymph node retrieval. Confound variables were age, BMI, number of adjuvant treatment and number of recurrence and these confound variables were corrected to compare the results according to the surgical method. The results showed significant statistical differences between the two groups for all three parameters. Specifically, the postoperative hospital discharge and total hospital charge were lower in the RSSRH group whereas lymph node retrieval was larger in the RMPRH group (*p* < 0.001).

**TABLE 4:** Comparison of RSSRH and RMPRH in cervical cancer by ANCOVA test (total hospital charge)

Source	Sum of squares	*F* value	*p*
Method	98 1503.0	106.15	<0.001
Age	8384.2	0.91	0.3433
Body mass index	5669.8	0.61	0.4354
Adjuvant therapy	26 908.1	2.91	0.0911
Recurrence	6996.9	0.76	0.3864

Abbreviations: ANCOVA, analysis of covariance; BMI, body mass index; RMPRH: robotic multiport radical hysterectomy; RSSRH: robotic single‐site radical hysterectomy.

**TABLE 5:** Comparison of RSSRH and RMPRH in cervical cancer by ANCOVA test (hospital discharge)

Source	Sum of squares	*F* value	*p*
Method	420.2	19.12	<0.001
Age	35.0	1.59	0.3433
Body mass index	44.1	2.01	0.1598
Adjuvant therapy	52.0	2.37	0.1271
Recurrence	46.5	2.12	0.1489

Abbreviations: ANCOVA, analysis of covariance; BMI, body mass index; RMPRH: robotic multiport radical hysterectomy; RSSRH: robotic single‐site radical hysterectomy.

**TABLE 6:** Comparison of RSSRH and RMPRH in cervical cancer by ANCOVA test (lymph node retrieval)

Source	Sum of squares	*F* value	*p*
Method	867.5	19.12	<0.001
Age	39.1	0.73	0.396
Body mass index	3.0	0.06	0.8124
Adjuvant therapy	30.1	0.56	0.4562
Recurrence	5.7	2.12	0.7452

Abbreviations: ANCOVA, analysis of covariance; BMI, body mass index; RMPRH: robotic multiport radical hysterectomy; RSSRH: robotic single‐site radical hysterectomy.

## 4 DISCUSSION

Compared to the laparoscopic single‐site surgery, the RSSS method provides to the operator an easier manipulation and enhanced approach possible at the operation field. The robot allows the surgeon to overcome technical problems including avoidance of tremors, a three‐dimensional vision and a precise control of the instruments to a complete 360°.^10^ While, the multiport robot technique produces optimal surgical results but leaves the patients with multiple scars. Fagotti et al.^14^ indicated that surgical scars may not represent a ‘cosmetic problem’, but rather a reflection of the impact that body image might have in each patient on the memory and experience of having cancer. The single‐site access (‘scar‐free surgery’) provides a solution to this problem by performing a minimal incision through the umbilicus access alone. In the gynaecological field, RSSS has been performed not only in benign conditions but also in malignant conditions since its FDA approval in 2013. Several studies have reported that RSSS is safe and feasible for benign and malignant surgery. However, most of the reported gynaecological cancers were endometrial cancers, and only a few studies have reported results on the use of the RSSS methods in patients with cervical cancer.^15^, ^16^, ^17^, ^18^

Hence, we have applied the RSSS method with pelvic node dissection to low‐risk cases with gynaecologic malignancies, such as early stage cervical cancer. In our study, RSSRH showed similar or better intraoperative and postoperative results than RMPRH, except for lymph node retrieval. There was no difference in estimated blood loss, blood transfusion, total operation time, haemoglobin change and overall complications, which is an indicator of the safety of the surgical method. In addition, there was an advantage in terms of postoperative hospital discharge and hospital charge. The ANCOVA test was applied to test statistical differences between the two groups. After correction for potential confounding factors linked to the performance of the two surgical methods in different periods and to the differences in sample size between the two groups. Overall, the results showed that the RSSRH method has better results in other parameters, except for lymph node retrieval.

However, there are several challenges when performing RSSRHs. First, the RSSRH may present high technical difficulties for the operator. In our centre, we started performing RSSRH in 2015, but our centre has performed since 2013 more than 300 cases of RSSS in benign and other gynaecologic malignant conditions. The notable surgical experience derived from the performance of a high number of RSSS procedures may also be linked to operation time. This factor may explain why we observed a shorter time for the RSSRH method but no difference in operation time, but the same pattern was not seen for the RMPRH method. An important limitation of the RSSRH is that there is less lymph node retrieval compared RMPRH procedures. This result suggests a limitation in accessing the retroperitoneal space for PLND compared to multiport settings, even though surgeons are provided with a wider range of motion with wristed instruments in the single‐port setting acquired with robotic technology. More importantly, this result can affect the oncologic outcomes of patients following radical hysterectomy. In particular, disease‐free survival and overall survival of MIS were inferior compared to laparotomy in the LACC trial and therefore a cautious application of MIS procedures has been recommended for the treatment of cervical cancer.^19^ In September 2019, the National Comprehensive Cancer Network (NCCN) recommended ‘Women should be carefully counseled about the short‐term versus long‐term outcomes and oncologic risks of the different surgical approach’ and Society of Gynecologic Oncology (SGO) also recommended ‘Gynaecologic oncologists should be aware of the continued emerging data on MIS for cervical cancer’.^20^, ^21^

Nevertheless, recent studies have reported that there is no difference in survival rate in early stage cervical cancer (less than 2 cm mass) between the laparotomy and MIS groups. Hence, it is necessary to carefully select the best surgical method to perform and also the most appropriate patient group to perform RSSRHs. In early stage cervical cancer (FIGO stage 1A1 with lympho‐vascular space invasion, 1A2 and 1B1), where lymph node metastasis is not suspected in preoperative imaging work up and tumour size is less than 2 cm, RSSRH may be considered to select carefully in consultation about emerging data on MIS with patients.

The aim of our study was to evaluate the safety and feasibility of the RSSRH operation involving lymph node dissection in early stage cervical cancer and to suggest surgical tips for node dissection using a robotic single‐site platform. We have showed that all RSSRH operations were accomplished successfully without additional port insertions, conversion to laparotomy or laparoscopy and intraoperative complications. We also showed that RSSRH for staging early cervical cancer is not inferior to RMPRH in terms of surgical outcomes and it may better result in terms of hospital charge and hospital stays. In addition, although the data were small, we confirmed that there was little difference for oncological outcomes between the two groups in patients with early lesions below FIGO stage IB1. However, this study has important limitations including the difference in sample size between the two groups (RSSRH: 20 vs. RMPRH: 42) and the difference in experience in performing the surgical procedures (i.e., RMPRH began in 2012, while RSSRH in 2015). As mentioned above, our centre had no difficulty in performing RSSRH procedures, since RSSS procedures were performed since 2013 for other benign and malignant conditions and, therefore, the operation time and hospital discharge appeared to be better for RSSRH. Based on our results, the RSSRH operation for early stage cervical cancer was safe and feasible and it may represent a viable surgical option in a carefully selected number of patients.

## 5 CONCLUSIONS

This study has showed that the RSSRH procedure was safer, more feasible, cost‐effective and had better short‐term perioperative outcomes than RMPRH. This technique could also be used to train residents and surgical fellows in well‐selected cases, although long‐term rates of complications and postoperative radiotherapy or chemotherapy associated with the procedures need to be explored. Randomized trials are needed to determine whether robotic single‐site techniques may offer clinical advantages over conventional procedures.

## CONFLICT OF INTERESTS

The authors declare that there no conflict of interests.
